# Evaluation of leukocyte depletion of packed red blood cell units and impact on clinically observed transfusion reactions

**DOI:** 10.3389/fvets.2023.1217575

**Published:** 2023-09-28

**Authors:** Barbara Steblaj, Jasmin Galli, Paul Torgerson, Annette Kutter

**Affiliations:** ^1^Section of Anaesthesiology, Department of Clinical Diagnostics and Services, Vetsuisse Faculty, University of Zürich, Zürich, Switzerland; ^2^Institute of Veterinary Anatomy, Vetsuisse Faculty, University of Zürich, Zürich, Switzerland; ^3^Section of Epidemiology, Vetsuisse Faculty, University of Zürich, Zürich, Switzerland

**Keywords:** blood product, canine, erythrocytes, leukocyte reduction, outcome, survival

## Abstract

**Introduction:**

The aim of this retrospective study was to determine whether there is an association between leukoreduction of packed red blood cell (pRBC) units and reduction of clinically observed transfusion reactions (TR), particularly febrile non-haemolytic transfusion reactions (FNHTR), and better outcomes in dogs. Secondary aims were to evaluate the effects of other factors suspected to influence transfusion reaction frequency or survival, including crossmatching, use of immunosuppressive drugs, and age and number of the blood products being administered.

**Materials and methods:**

Medical data on dogs transfused with leukocyte-reduced (LR) and non-leukocyte-reduced (N-LR) pRBC units at the Animal Hospital Zürich, University of Zürich, Switzerland between January 1, 2007, and December 17, 2018 were searched. Before 2014, only N-LR blood were transfused. After 2014, both LR and N-LR blood were available.

**Results:**

A total of 339 canine patients were transfused with 413 pRBC units; 30.5% (126/413) were LR units and 69.5% (287/413) were N-LR. Data collected from medical records was analyzed using univariate and multivariate logistic regression. In the present study, TR occurred in 19.8% of pRBC units (25/126) with LR and in 17.7% (51/287) of pRBC with N-LR; *p* > 0.05. FNHTR occurred in 6.3% of pRBC units (8/126) with LR and in 4.5% (13/287) of those with N-LR; *p* > 0.05. There was no correlation between the occurrence of TR and discharge from hospital (*p* > 0.05). Crossmatching, immunosuppressive therapy, and age of the blood product were not associated with the frequency of TR; *p* > 0.05 for all. The duration of survival days was not related to the number of transfusions dogs received.

**Discussion:**

In the present study, the leukocyte-depletion of transfused pRBC units was not associated with fewer TR nor to fewer FNHTR compared to N-LR units. Discharge of dogs from hospital was not dependent on the occurrence of TR.

## 1. Introduction

In veterinary medicine, transfusion of blood products, such as packed red blood cell (pRBC) units, has become an essential part of the treatment of anemia (1, 2). Due to risk of acute or delayed immunological or non-immunological reactions (2, 3), transfusion of blood products should be carefully considered before it is performed (4–8).

In humans, leukocytes transfused with pRBC units are known to trigger an inflammatory process in the recipient through the production of cytokines (9). This can result in febrile non-hemolytic transfusion reaction (FNHTR). A FNHTR in humans is characterized by a fever of at least 38°C and an increase of at least 1°C from pre-transfusion values during or within 4 h after the end of a transfusion, or by rigors/chills during the same period and the absence of other causes (10). This reaction is thought to be triggered by an interaction between the recipient's cytotoxic antibodies and the human leukocyte antigen and/or WBC-specific antigens on donor's WBCs. The formation of antigen-antibody complexes leads to complement binding and the release of endogenous pyrogens. Stored cytokines and inflammatory mediators that are transfused mediate a direct immunological response. Platelet storage, which involves continued production and release of biologically active cytokines by residual WBCs, may result in a similar clinical outcome (9). Several studies in human medicine have shown that transfusion of leucocyte-reduced (LR) blood products reduces the incidence of FNHTR (9, 11, 12). However, a Cochrane review in humans failed to demonstrate any beneficial or detrimental effects of LR blood transfusion products on outcome (13).

Recently, Transfusion Reaction Small Animal Consensus Statement (TRACS) guidelines have provided a definition, diagnosis, and treatment of transfusion reactions (TR) in small animals. A FNHTR in small animals is defined as an acute non-immunological or immunological reaction characterized by a temperature > 39°C and a temperature rise of >1°C from pre-transfusion body temperature during or within 4 h of the end of a transfusion, where external warming, underlying patient infection, acute hemolytic transfusion reaction (AHTR), transfusion related acute lung injury (TRALI), and transfusion transmitted infection (TTI) have been ruled out (7). A study in dogs showed that several inflammatory parameters such as leukocyte count, fibrinogen, and serum C-reactive protein concentration were lower when autologous LR blood was transfused back to the donor compared to N-LR blood (14). Interestingly, in another study, transfusion of autologous LR blood to dogs did not result in less inflammation or better clinical outcome compared to N-LR. The same study, however, showed that only the prolonged storage of the blood product led to an increase in inflammation (15). Another study in dogs found no difference in the degree of inflammation between LR and N-LR blood transfusion, although the study may have been underpowered (16). The literature on outcomes following transfusion of LR pRBC is sparse. A smaller prospective clinical study showed no beneficial effect of LR on occurrence of TRs or survival (17), while a larger retrospective study showed lower incidence of FNHTRs with LR pRBC units (18). Whether a reduction in leukocytes in blood transfusion product would result in fewer clinically observed FNHTR or other TRs remains unclear. In addition to FNHTR, other TRs such as respiratory reactions [Transfusion-Associated Dyspnea (TAD), Transfusion-Associated Cardiovascular Overload (TACO), and TRALI], allergic reactions, AHTR, TTI, Hypocalcemia/Citrate toxicity, Transfusion-Related Hyperammonemia, or Hypotensive Transfusion Reactions (HyTR) and Post-transfusion purpura have been recently defined in TRACS guidelines (7).

While LR may reduce the incidence of FNHTR, the incidence of acute immunological TR could also be reduced by crossmatching, use of pharmacological intervention (immunosuppressive therapy), or shorter storage of blood products. Crossmatching prior to first transfusion in dogs has been shown to result in higher packed cell volume (PCV) levels than when no crossmatching was performed (19). Whether pharmacological intervention reduces the incidence of adverse acute TR in dogs remains unclear (20); however pharmacological interventions have not been shown to prevent FNHTR in humans (21). Callan et al. (15) demonstrated a stronger inflammatory response in dogs receiving older blood products. The literature on the association between TR/survival and the amount of blood units transfused is sparse (18).

The primary aim of this retrospective study was to compare the frequency of FNHTRs and other acute TR between LR and N-LR pRBC units administered to canine patients and to compare patient outcomes. Secondary aims were to evaluate the effects of other factors suspected to influence transfusion reaction frequency and dogs' survival, including crossmatching, use of pharmacological interventions (immunosuppressive drugs), and age and number of the blood products being administered.

## 2. Materials and methods

### 2.1. Animals and data collection

The medical records of canine patients who had received a pRBC transfusion at the Animal Hospital Zürich, University of Zürich, Switzerland between January 1, 2007 and December 17, 2018 were searched. Data collected included the signalment of the patient (breed, age, sex, and weight), reason for blood transfusion (blood loss, hemolysis, and lack of production based on clinical diagnosis at time of transfusion), whether a TR occurred, information about the blood product (LR, donor blood type, and age of blood), whether a crossmatch was performed before the transfusion, whether the patient received immunosuppressive drugs before the transfusion or received previous blood transfusions, the PCV level before and after the transfusion, the acute outcome (discharge, euthanasia, or death), and the patient's survival after 30 days after the first transfusion. During the transfusion, dogs' heart rate, respiratory rate, blood pressure, temperature, mentation, and potential signs of TR such as swelling, vomiting, diarrhea, pruritus, and urticaria were monitored. These parameters were recorded at baseline, at 5, 10, 20, and 30 min, and at 1, 1.5, 2, 2.5, 3, and 4 h after the start of transfusion. Of note, all the medications were stopped during administration of blood transfusion.

We included dogs that had received pRBC units between January 1, 2007 and December 17, 2018. Dogs, that lacked information on the type of blood transfusion product (LR or N-LR) or whether TR occurred were excluded from analysis. A history of previous transfusion was recorded. Cases of dogs receiving multiple transfusions from the same donor on the same day were considered one transfusion, whereas transfusions from the same donor on different days and transfusions from different donors on the same or different days were considered multiple transfusions.

### 2.2. Definition of transfusion reactions

A FNHTR was defined as a rise in body temperature above 39 and 1°C or more within 4 h of the start of the transfusion. This increase was only considered if the baseline temperature was not in the hypothermic range, i.e., below 37.5°C, as patients with hypothermia are generally warmed up with warming devices in our institution. In addition, we defined patients with FNHTR as such only if they had no other condition that could explain the fever and had no hemolysis. A specific type of TR was diagnosed based on the recently published veterinary TRACS guidelines (7). We searched transfusion monitoring logs, medical records, and kennel charts to determine the type of TR and if TR occurred within 24 h of starting the transfusion. We defined as Other TR all TR that occurred excluding FNHTR. All TR included FNHTR as well as Other TR. Suspected transfusion reactions based on clinical signs that occurred during transfusion but were not present prior to transfusion and could not be linked to a medical condition or assigned with certainty to one of the definitions in the recently published TRACS guidelines were defined as Uncategorized TR (Table 1).

**Table 1:** Reasons for blood transfusions.

**Reason for blood transfusion**	**Non-leukocyte-depleted pRBC units administered**	**Leukocyte-depleted pRBC units administered**
**Number of units**	***n*** = **287**	***n*** = **126**
Blood loss	168/244 (68.9%)	76/244 (31.1%)
Hemolysis	81/114 (71%)	33/114 (29%)
Lack of production	28/35 (80%)	7/35 (20%)
Reason unknown	10	10

### 2.3. Immunosuppressive therapy and PCV

Immunosuppressive drugs included steroids, azathioprine, cyclosporine, mycophenolate, and leflunomide. They were included in the statistical analysis if they had been administered/started before blood transfusion. The PCV values considered for this study were always the last value measured before transfusion and the first value measured after the transfusion.

### 2.4. Blood collection and processing

Non-LR blood was collected in a 3-bag system (CompoFlex^®^, Fresenius Kabi, Germany) without a leukocyte reduction filter, while LR blood was collected in a 4-bag system (CompoSelect^®^, Fresenius Kabi, Germany) with an integrated leukocyte reduction filter (<1 × 10^6^ U leucocytes left after filtration of whole blood unit). The blood was left at room temperature for at least 40 min before being filtered and centrifuged (Centrifuge Rotixa 50 RS Typ 4910, Hettich, Switzerland) at 5,000 g for 10 min at 4°C to separate into pRBC and plasma. Each pRBC unit was labeled accordingly as LR or N-LR blood. Blood was stored vertically in a refrigerator at 4°C. Before 2014, only N-LR blood was available; after 2014, both LR and N-LR blood were available. The clinician could choose between the units that were available at a given time, usually choosing units that expired first. From 2007 to 2011, the blood typing method used at our institution to test recipients of the blood in an emergency situation was blood typing cards, while more recently immunochromatographic cartridges have been used (Alvedia, Quick Test DEA 1.1, France). Donors and recipients of the blood in non-emergency situation were blood typed in the faculty laboratory using an erythrocyte agglutination test. Crossmatching was performed either in the faculty laboratory using an erythrocyte agglutination test or in an emergency with a commercially available canine crossmatching test (Quick Test XM, Alvedia, France).

### 2.5. Statistical analysis

Descriptive statistics were performed. The frequencies of sex, breed, blood groups, reasons for transfusion, outcomes, survival, blood group of transfused units, number of transfusions, and TR were calculated. All statistical analyses were performed using the statistical computing software R version 3.6.2. The normality of the data distribution was assessed using a Shapiro-Wilk test. Data are reported as mean ± SD for normally distributed data and median and range (minimum-maximum) for non-normally distributed data. A Fisher exact test was used to compare the occurrence of TR between LR and N-LR blood. A Kruskal-Wallis Chi-square test and a Mann-Whitney test were used for non-parametric data. Bonferroni correction was used when indicated. Multivariable logistic regression was used to investigate whether leukocyte-depletion, crossmatch, and immunosuppressive drugs are associated with the occurrence of transfusion-related complications. Information on dose and time of immunosuppressive drugs was frequently not reported. Thus, we separately analyzed the association of TRs with LR and N-LR blood excluding patients that received immunosuppressive therapy. Logistic regression was used to examine risk factors such as breed, sex, age, weight, age of the blood, multiple transfusions, and reason for pRBC transfusion for the occurrence of TR. The impact of TR on acute outcome and survival at 30 days and the value of PCV before and after transfusion were analyzed using logistic regression. Values of *p* < 0.05 were considered significant.

## 3. Results

### 3.1. Descriptive data

**Figure 1:**
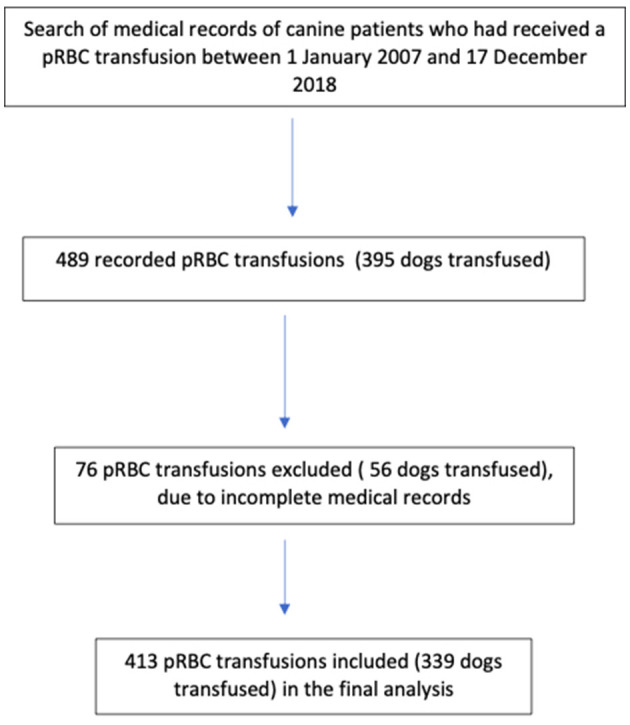
Selection of patients.

Of 489 transfusions recorded, five units did not have records of whether they were LR or N-LR and 71 lacked a record of whether a TR occurred. Of the 395 dogs initially included, 56 dogs that had received 76 units of pRBC were excluded from the analysis as their incomplete medical records could not determine whether they had suffered from TR or whether the blood product they had received had been LR. The final analysis included 339 dogs that had received 413 blood products (Figure 1). There were 170 males (50.1%), 84 intact (49.4%) and 86 neutered (50.6%), and 169 females (49.9%), 58 intact (34.3%) and 111 neutered (65.7%), included. The age of all dogs was 7.8 ± 3.6 years (mean ± standard deviation); (N-LR: 7.7 ± 3.6 years, LR: 7.68 ± 3.6 years) and the body weight was 20.7 ± 13.7 kg (N-LR: 22.58 ± 13.37 kg, LR: 19.46 ± 12.92 kg). The study included 78 different breeds and the largest group consisted of mixed breed dogs (80/339, 23.6%). The breed of 15 dogs was not reported (4.4%). Breed, sex, age, or weight were not significantly related to TR in this study using univariate logistic regression (*p* > 0.05).

### 3.2. Blood group and age of the blood

Blood group dog erythrocyte antigen (DEA) 1 positive was present in 196 dogs (57.8%), blood group DEA 1 negative was present in 122 dogs (36%), and the blood group of 21 dogs was not recorded (6.2%). Of the 413 pRBC units transfused, 212 units were DEA 1 positive (51.3%), 198 units were DEA 1 negative (48%), and blood group was not recorded for 3 units of pRBC (0.7%). DEA 1 negative dogs were transfused with DEA 1 negative blood, while DEA 1 positive dogs were transfused with DEA 1 positive or negative blood. Only one DEA negative dog was transfused with DEA positive blood.

The age of the N-LR blood was 23 days (0–44 days) and the LR blood was 18 days (0–46 days) (*p* = 0.0035). The age of the blood was not associated with occurrence of FNHTR [Kruskal-Wallis Chi-square = 2.2895, degrees of freedom (*df*) = 1, *p*-value > 0.05] or other TR (Kruskal-Wallis Chi-square = 0.074578, *df* = 1, *p*-value > 0.05). There was no difference in occurrence of TR between blood age younger or older than 28 days (*p*-value > 0.05). There was also no difference in occurrence of TR between blood age younger or older than 35 days.

### 3.3. Indications for transfusion

The main indication for transfusion was blood loss in 213/339 (62.8%) dogs, followed by hemolysis in 85/339 dogs (25.1%), lack of production in 25/339 dogs (7.4%), and in 16/339 (4.7%) cases it could not be assigned to any of these groups. Of the 244 units of packed red blood cells transfused to dogs with blood loss, 168/244 units (68.9%) were N-LR and 76/244 units (31.1%) were LR. One hundred and fourteen units were transfused to patients with hemolysis, 81/114 units (71%) were N-LR, and 33/114 units (29%) were LR. Finally, of the 35 units transfused to canine patients with an underlying disease resulting in lack of red blood cell production, 28/35 units (80%) were N-LR and 7/35 units (20%) were LR (Table 1).

### 3.4. Packed cell volume before and after the transfusion

The PCV before transfusion was 14% (6–32%) in dogs who received N-LR and 12% (6–29%) in dogs who received LR blood (*p* > 0.05). After the transfusion, PCV was 22% (7–45%) in dogs who received N-LR and 21% (10–44%) in dogs who received LR blood (*p* > 0.05). PCV change (PCV post-transfusion—pre-transfusion) in dogs receiving either LR or N-LR blood was not significantly different (*p* > 0.05). In addition, there was no association between age of the blood and PCV after the transfusion (*p* > 0.05).

### 3.5. Transfusion reactions

No correlation was found between leukocyte reduction and occurrence of FNHTR or other TR (Table 2). Leukocyte depletion of blood products was not associated with fewer FNHTR (*p* > 0.05). Transfusion reaction occurred with 76/413 pRBC units transfused (Table 2) and FNHTR occurred in 21/76 cases, representing 27.6% of all TRs that occurred. The incidence of TR with LR and N-LR pRBC units is shown in Table 2. FNHTR occurred in 16/319 (5%) dogs with first transfusion and in 5/93 (5.3%) dogs with multiple transfusions; there was no association between occurrence of FNHTR and number of transfusions, i.e., whether the dog received first transfusion or consecutive transfusions (*p* > 0.05). In addition, multiple transfusions were not identified as an increased risk factor for TR (*p* > 0.05). There was no significant difference in the incidence of TR before 2014 (the year we started leukoreduction of blood products) and after 2014 (*p* > 0.05). Multivariable logistic regression showed no association between cause of transfusion (hemolysis, blood loss, and lack of production) and occurrence of TR (*p* > 0.05). Fever resolved in two cases on its own, in 17 cases after steroid administration, in one case after metamizole administration, and in one case data was not available. Dogs that received steroids were already on them due to underlying disease.

**Table 2:** Incidence of total transfusion reactions, including febrile non-hemolytic transfusion reactions (FNHTR) and other TR with leukocyte-depleted and non-leukocyte-depleted pRBC units, in 339 canine patients administered 413 packed red blood cell (pRBC) units.

**pRBC units given**	**Total 413 units (100%)**		
	**Non-leukocyte-depleted pRBC 287/413 (69.5%)**	**Leukocyte-depleted pRBC 126/413 (30.5%)**	***p*** **-value**
	**76/413 (18.4%)**		
Total transfusion reactions	51/287 (17.7%)	25/126 (19.8%)	*p* > 0.05
	**21/413 (5.1%)**		
FNHTR	13/287 (4.5%)	8/126 (6.3%)	*p* > 0.05
	**55/413 (13.3%)**		
Other transfusion reactions	38/287 (13.2%)	17/126 (13.5%)	^**^p > 0.05
AHTR	4/287 (1.4%)	0/126 (0%)	
HyTR	4/287 (1.4%)	4/126 (3.2%)	
Allergic TR	3/287 (1%)	2/126 (1.6%)	
Respiratory TR	5/287 (1.7%)	1/126 (0.08%)	
Uncategorized^*^ TR	22/287 (7.6%)	10/126 (8%)	

pRBC, packed red blood cell; FNHTR, febrile non-hemolytic transfusion reaction; AHTR, acute hemolytic transfusion reactions; HyTr, hypotensive transfusion reactions.

Asterisk marks the reactions that could not be assigned to any definition of transfusion reaction according to TRACS guidelines.

^**^p-values for each category were > 0.05.

All TR includes FNHTR and other TR (i.e., AHTR, HyTR, Allergic, respiratory, and uncategorized TR).

### 3.6. Pretransfusion crossmatch and immunosuppressive therapy

In 43/413 (10.4%), cases, crossmatching was performed before transfusion started, in 306/413 (74.1%) cases no crossmatching was performed, and in 64/413 (15.5%) cases it was not reported if crossmatching was performed. There were eight dogs in which a crossmatch was performed before the first transfusion, in 22 dogs before the second transfusion, in 10 dogs before the third transfusion, in two dogs before the fourth transfusion and in one dog before the fifth transfusion. Eight canine patients (8/43, 18.6%) who had been crossmatched suffered from TR. Performing major crossmatching before starting a transfusion was not associated with significantly fewer TR (*p* > 0.05). Acute hemolytic transfusion reaction occurred in 1/43 (2.3%) crossmatched cases, 2/306 (0.6%) non-crossmatched cases, and 1/64 (1.5%) cases where it was not reported if crossmatching has been performed or not. Crossmatching was not associated with lower occurrence of AHTR (*p* > 0.05).

Of 413 units administered, immunosuppressive therapy was administered in 149 (36.1%) cases, no immunosuppressive therapy was administered in 166 (40.2%) cases, and in 98 (23.7%) cases it was not recorded whether immunosuppressive therapy was administered or not. Dogs receiving immunosuppressive therapy were usually on this therapy due to immune-mediated hemolytic anemia (IMHA). Hemolysis was a reason for transfusion in 109 patients, of which 69/109 (63.3%) received immunosuppressive drugs, 15/109 (13.7%) did not receive immunosuppressive drugs, and there was a lack of record for 25/109 (22.9%) patients. Twenty-one patients (21/69, 30.4%) who received immunosuppressive drugs for hemolysis of erythrocytes also developed TR, 48/69 (69.5%) patients received immunosuppressive drugs but showed no signs of TR, 6/15 (40%) patients received no immunosuppressive drugs and developed TR, and 9/15 (60%) patients received neither immunosuppressive drugs nor showed signs of TR. Administration of immunosuppressive drugs was not associated with a lower risk of total TR (*p* > 0.05). As the dose of immunosuppressive drugs was not always known, we also analyzed association of TR with LR and N-LR blood excluding patients that received immunosuppressive drugs; there was no significant difference with occurrence of FNHTR, AHTR, HyTR, allergic, respiratory TR, or uncategorized TR (*p* > 0.05 for all TR) with LR or N-LR blood.

### 3.7. Outcome and survival

The outcome in dogs receiving N-LR or LR pRBC units was not significantly different (Kruskal-Wallis Chi-square = 5.319, *df* = 2, *p* > 0.05) and is shown in Table 3. There was no correlation between the occurrence of transfusion reactions and the discharge of the dog (*p* > 0.05).

**Table 3:** Outcome of 339 dogs receiving either leukocyte-depleted or non-leukocyte-depleted packed red blood cell units.

	**Non-leukocyte-depleted pRBC**	**Leukocyte-depleted pRBC**	**Total number of dogs**	***P*-value**
**Number of dogs**	***n*** = **241**	***n*** = **98**	***n*** = **339**	
Discharged	153/241 (63.5%)	58/98 (59.2%)	211/339 (62.2%)	*p* > 0.05
Euthanized	51/241 (21.1%)	31/98 (31.6%)	82/339 (24.2%)	
Died	37/241 (15.4%)	9/98 (9.2%)	46/339 (13.6%)	

pRBC, packed red blood cells.

In 158 dogs receiving one transfusion, the median survival was 2 days (0–1, 419 days), in 44 dogs receiving two transfusions, median survival was 3.5 days (0–517 days), in seven dogs receiving three transfusions, it was 0 days (0–5 days), and in two dogs receiving four transfusions, median survival was 1 day (1–1 days). The survival days of the dogs were not related to the number of transfusions received (Kruskal-Wallis Chi-square = 0.7937, *df* = 1, *p* > 0.05). Survival of dogs receiving either N-LR or LR pRBC units was also not significantly different (Kruskal-Wallis Chi-square = 0.6403, *df* = 2, *p* > 0.05) and is shown in Table 4. Multivariable analysis showed that neither the pre-transfusion PCV nor the post-transfusion PCV value were significantly associated with survival 30 days post-transfusion (*p* > 0.05).

**Table 4:** Follow-up of 211 dogs discharged after receiving either leukocyte-depleted or non-leukocyte-depleted packed red blood cell (pRBC) units.

**Survival**	**Non-leukocyte depleted PRBC**	**Leukocyte-depleted PRBC**	**Total number of dogs**	***p*-value**
**Number of dogs**	***n*** = **153**	***n*** = **58**	***n*** = **211**	
Alive after 30 days	80/153 (52.3%)	27/58 (46.6%)	107/211 (50.2%)	*p* > 0.05
Euthanized or died	11/153 (7.2%)	4/58 (6.8%)	15/211 (7.1%)	
Lost to follow-up	62/153 (40.5%)	27/58 (46.6%)	90/211 (42.7%)	

pRBC, packed red blood cells.

## 4. Discussion

In this study the diverse patients were a good representation of a diverse population with a similar proportion of females (intact and neutered) and males (intact and neutered), a wide range of young to old dogs, and many different small, medium, and large dog breeds.

### 4.1. Descriptive data

There were more DEA 1 positive patients (57.9%) than DEA 1 negative patients (36%), which fits within the expected range of DEA 1 frequency of 33–60% found in previous studies (19–21). However, only 51.3% of transfused blood was DEA 1 positive and 48% was DEA 1 negative. This can be explained by the fact that it is accepted practice to administer DEA 1 negative blood to DEA positive recipients. Only one DEA negative dog received DEA positive blood (due to unavailability of DEA negative blood), while DEA negative dogs received DEA negative blood and DEA positive dogs received either DEA positive or DEA negative blood. This means that, with the exception of one dog, all dogs were given appropriate blood according to their blood group.

### 4.2. Reason for transfusion

Hemolysis as a reason for red blood cell transfusion has previously been associated with an increased risk of TR in dogs (20). In immune-mediated diseases such as IMHA, autoantibodies that bind to antigens on the outer red blood cell membrane lead to activation of complement and subsequent destruction of these red blood cells (22). However, the results of our study show that patients with hemolysis are not more likely to develop TR.

### 4.3. Age of the blood

A potential risk factor for the development of TR advocated by several studies is the age of the transfused blood product (6, 20, 23). The age of the blood transfusion products was significantly different between N-LR and LR blood, however we could not demonstrate an association with incidence of TR and the age of the blood product. An experimental study by Callan et al. (15) using autologous blood has shown that a higher age of the blood product (28 days) triggers an inflammatory response independent of leukoreduction without clinically observed TR in dogs either with or without leukoreduction. Our analysis also could not demonstrate statistical difference in clinically observed TR between either blood age above 28 or 35 days. In our study, the storage time of LR blood was shorter than with N-LR blood; despite that there was no difference in incidence of TR between LR and N-LR PRBC units. These findings correlate well with the results of Callan.

### 4.4. Effect of leukoreduction on incidence of transfusion reactions

In this study, the incidence of FNHTR and other TR in canine patients receiving LR pRBC units did not differ from those receiving the N-LR pRBC units.

The incidence rate for overall TR in canine transfusions performed with LR pRBC was statistically not lower (19.8%) than the incidence rate for TR in transfusions performed with N-LR pRBC (17.7%). The overall incidence rate for TR of 18.4% in our study is comparable to the incidence of TR in previously conducted studies in canine transfusion medicine, which ranged from 3.3 to 28% (5, 6, 20).

Correct identification of TR is difficult, and inflammatory reactions or other adverse effects due to transfusion may be incorrectly attributed to ongoing inflammatory processes based on underlying diseases, which is one of the reasons why the percentages reported in our study may be an under- or overestimation of actual cases of TR (13). As the monitoring protocol was only completed up to 4 h after the start of transfusion, some TRs may have been missed as they may have occurred later, were not recognized as such, or were not reported in the medical records. Due to the retrospective nature of this study, some adverse reactions could not be categorized into a single TRACS-determined TR definition.

Like the overall incidence of TR, the incidence of FNHTR did not differ between groups, with 6.3% of canine patients receiving LR pRBCs compared with 4.5% in dogs not receiving LR pRBC. In the literature, the incidence of FNHTR in dogs has been reported to be 1.3–24.2% (15, 20, 23, 24). A recent prospective study with an incidence of 7.7% (17) is similar to our study (5.1%). In human medicine, the classification of FNHTR often includes nausea and vomiting (25). However, we followed TRACS guidelines and only included dogs in which the only clinical sign was an increase in body temperature above 39°C and an increase of at least 1°C. If we had included dogs that also developed nausea and vomiting, more dogs would have been diagnosed with FNHTR. In humans, it is reported that most FNHTRs occur during the first transfusion (26). We were unable to demonstrate this in our study. This could be due to a lack of dog natural antibodies against foreign erythrocyte antigen before the first contact with it.

Based on the results of our study, we cannot determine whether leukoreduction has beneficial or harmful effects. McMichael et al. found a significant increase in leukocyte count, fibrinogen level, and C-reactive protein when healthy dogs were transfused with autologous N-LR blood (14). In contrast to McMichael's study (14) which evaluated autotransfusion, Bosch Lozano et al. (27) did not demonstrate beneficial effects of LR pRBC in critically ill dogs. However, 24 h after transfusion, leukocyte counts were higher in dogs that received N-LR pRBC units compared to LR pRBC units. This transfusion-induced inflammation could be of clinical importance in dogs with ongoing inflammatory processes, as it may negatively affect patient morbidity by increasing metabolic demand and oxygen consumption (28). A small prospective study by Radulescu et al. (17) also found no benefit of transfusion of LR blood on the incidence of clinically observed TR. In addition, TRACS guidelines indicate that there is currently insufficient evidence for or against leukoreduction (LR) to prevent or reduce any type of TR in veterinary medicine. Since there is evidence in human studies that LR reduces the rate of FNHTR, they recommend that this be considered. On the contrary, a large retrospective study by Davidow et al. (18) involving 455 dogs found a reduced rate of TR in dogs receiving LR blood units. In contrast to this study, the Davidow study did not use the TRACS TR definitions as it was completed prior to their development. Additionally, we could not demonstrate a reduction in incidence of TR with LR of pRBC units in our study. It is well known from human medicine that most FNHTR are benign events (29), whereas transfused leukocytes could harm the recipient as a result of the modulation of the recipient's immune system (30). The costs and benefits of leukoreduction should also be considered. The study by Trinder et al. (31) could partially answer these questions by demonstrating that the volume and time needed for leukoreduction of blood is not significantly different from the N-LR blood processing procedure.

It is known that WBC have shorter life spans and their number decreases quicker in blood product compared to RBC. Therefore, one might assume that leukoreduction is of no benefit. However, we believe that this does not prevent cytokines and other potential immune triggering substances to be accumulated within blood product. Therefore, leukoreduction should be performed before WBC have an opportunity to release immune triggering substances in the blood transfusion product. Additionally, WBC poses WBC- specific antigen, which might cause an immune-mediated reaction in a recipient of blood transfusion product (9). Additionally, some leucocyte chemoreceptors might increase while others decrease in stored blood products, leading to an inflammatory response in the recipient (32). Although most FNHTR are known to be harmless in humans and resolve on their own, we have little data to confirm that they are harmless in critically ill dogs. Even if there is no clinically observed reaction, there might still be immune reaction as shown by Callan. Therefore, it might be that in already critical patients with compromised immune and organ systems, additional inflammation could influence the outcome. An extremely large study would likely be needed to confirm or omit this.

### 4.5. Crossmatching and immunosuppressive therapy

Crossmatching (major compatibility testing) was not associated with a lower risk of AHTR with the data collected in this study. However, only the blood of 43 patients was crossmatched before transfusion. This is usually performed at our institution if the dog had previously received other transfusions or if the transfusion history is unknown. Because crossmatching tests the compatibility of the donor's red blood cells antigens and the recipient's antibodies, the use of this method may reduce the incidence of AHTR (4, 20). It could be that crossmatching was not associated with a lower incidence of AHTR, since blood recipients who do not match the donor's blood do not receive it anyway.

Bruce et al. (20) study also showed no protective role of corticosteroids against TR. A Cochrane study (21) in humans also found no benefit of pre-treatment with corticosteroids in reducing the incidence of TR. Unfortunately, we cannot report at what time the immunosuppressive drugs were started/administered prior to admission to our clinic, as this information was mostly not recorded.

### 4.6. Outcome and survival

The present study did not demonstrate an increased risk of TR associated with multiple red blood cell transfusions. This correlates well with Bruce's study (20). We also could not demonstrate an association between number of transfusions and survival. Bosch Lozano et al. reported similar survival rates to discharge with LR and N-LR pRBC unit being transfused to critically ill dogs (27). Whether leukocyte reduction can prevent death is inconsistent (33).

In our study, for the 82 dogs that were euthanized, the reason for euthanasia was not reported. Dogs that were euthanized due to owner's lack of financial means may have influenced the results of this study. Whether TR occurred or not was not associated with hospital discharge.

In addition, the survival rate after 30 days is not known for 90 dogs that were lost for follow-up. This could have greatly influenced the data. Many dogs lost to follow-up did not require further treatment or continued treatment at a private veterinarian and may have been still alive 30 days after discharge from our facility. Therefore, 50.2% of dogs that were alive after 30 days might be an underestimate.

### 4.7. Limitations

This study has a number of limitations due to its retrospective nature. The study was conducted over a long period of time during which many standard operating procedures in canine transfusion medicine at the Animal Hospital Zürich have changed and improved. Since leukocyte depletion of pRBC was only introduced in the Animal Hospital Zürich in 2014, all LR units were transfused to dogs under surveillance of multiparametric anesthesia monitoring compared to the previous year, so more TRs might have been identified as such. Despite more strict and objective monitoring after 2014 using a multiparametric monitor, we could not demonstrate a significant difference in the occurrence of TR before and after 2014. In addition, only 30.5% of the transfused units in this study were LR and 69.5% were not LR. It is also important to note that strict monitoring was only applied for the first 4 h so some immunomodulatory reactions might have been missed.

This study is unable to demonstrate a clinical benefit of leukocyte depletion of pRBC units in canine transfusion medicine. However, if leukocyte depletion had been of great importance, one would have expected at least a small trend to be documented with this data set of 339 dogs.

Ours and Davidow's study, while large, are still relatively small given the number of FNHTR. On the human side, the association is only found when looking at extremely large studies. An extremely large prospective study is likely needed to overcome the limitations of the current study. Results of our study can be used as a way to calculate the study size required.

## 5. Conclusion and clinical relevance

This study showed no difference between the occurrence of TR (neither FNHTR nor other types of TR) after the use of LR and N-LR pRBC units. The occurrence of TR in dogs was not associated with the likelihood of being discharged.

## Data availability statement

The original contributions presented in the study are included in the article, further inquiries can be directed to the corresponding author.

## Ethics statement

Ethical approval was not required for the studies involving animals in accordance with the local legislation and institutional requirements because only medical records were searched and analyzed. Written informed consent was not obtained from the owners for the participation of their animals in this study because general written consent was obtained from clients, that data obtained during hospitalization can be analyzed for potential studies.

## Author contributions

BS, JG, and AK conceived and designed the study and collected, complied, and analyzed data. PT performed statistical analysis and complied data. BS, JG, PT, and AK drafted and edited the manuscript. All authors contributed to the article and approved the submitted version.
